# Design, Optimization, and Experimental Evaluation of Slow Light Generated by π-Phase-Shifted Fiber Bragg Grating for Use in Sensing Applications

**DOI:** 10.3390/s24020340

**Published:** 2024-01-06

**Authors:** Matúš Vaňko, Ivan Glesk, Jarmila Müllerová, Jozef Dubovan, Milan Dado

**Affiliations:** 1 Department of Multimedia and Information-Communication Technologies, Faculty of Electrical Engineering and Information Technology, University of Žilina, Univerzitná 8215/1, 010 26 Žilina, Slovakia; matus.vanko@uniza.sk (M.V.); jozef.dubovan@uniza.sk (J.D.); milan.dado@uniza.sk (M.D.); 2 Electronic and Electrical Engineering Department, University of Strathclyde, 204 George Street, Glasgow G1 1XW, UK; 3 Institute of Aurel Stodola, Faculty of Electrical Engineering and Information Technology, University of Žilina, Komenského 843, 031 01 Liptovský Mikuláš, Slovakia; jarmila.mullerova@uniza.sk

**Keywords:** π-phase-shifted fiber Bragg grating, slow light generation, group delay, optical fiber sensors

## Abstract

This paper describes design, theoretical analysis, and experimental evaluation of a π-Phase-Shifted Fiber Bragg Grating (π-PSFBG) inscribed in the standard telecom fiber for slow light generation. At first, the grating was designed for its use in the reflection mode with a central wavelength of 1552 nm and a pass band width of less than 100 pm. The impact of fabrication imperfections was experimentally investigated and compared to model predictions. The optical spectra obtained experimentally show that the spectral region used for slow light generation is narrower (less than 10 pm), thus allowing for too-low levels of slow light optical-output power. In the next step, the optimization of the grating design was conducted to account for fabrication errors, to improve the grating’s spectral behavior and its temporal performance, and to widen the spectral interval for slow light generation in the grating’s transmission mode. The targeted central wavelength was 1553 nm. The π-PSFBG was then commercially fabricated, and the achieved parameters were experimentally investigated. For the region of (1551–1554) nm, a 15-fold increase in the grating’s pass band width was achieved. We have shown that a pair of retarded optical pulses were generated. The measured group delay was found to be ~10.5 ps (compared to 19 ps predicted by the model). The π-PSFBG operating in its transmission mode has the potential to operate as tunable delay line for applications in RF photonics, ultra-fast signal processing, and optical communications, where tunable high precision delay lines are highly desirable. The π-PSFBG can be designed and used for the generation of variable group delays from tens to hundreds of ps, depending on application needs.

## 1. Introduction

The development of a low cost and high accuracy instrumentation has been the main driver behind using the fiber optics in many areas. The use of fiber Bragg gratings (FBGs) in sensor applications have been extensively studied over past decades. Sensing with optical sensors is developing very rapidly, many fiber optics network elements used today can be found in light generation [1], demodulation [2], or used in optical delay lines [3]. Apart from a smaller size, the immunity to electromagnetic interference and the ability to measure multi-parameter phenomena, FBG sensors offer many advantages. Measuring the strain, temperature variations, acoustic emission, and magnetic field [4] are all already implemented in structural health monitoring or many biomedical applications [5]. FBG sensors based on slow light offer higher sensitivity, wider dynamic range, and better resolution in comparison to conventional FBG sensors. Their performance in applications has improved significantly, namely in terms of the sensitivity and dynamic range [6,7,8,9,10]. Strain and temperature sensing based on slow-light π-phase-shifted FBG (π-PSFBG) was also reported as promising [8].

Building on their potential, additional enhancement of FBG sensors sensitivity is possible with the use of slow light produced by a uniform phase shifted grating [10]. Light propagating through the region designed for multiple reflections will cause an effective decrease of the group velocity. Sensors designed to benefit from slow light take an advantage of the controlled light-matter interaction. Since the sensitivity of a phase sensor to external perturbations is inversely proportional to a group velocity in the slow-light region, the sensitivity of a π-PSFBG is significantly enhanced over the sensitivity of traditional FBG sensors operated around the Bragg wavelength [6]. The influence of slow-light grating parameters, on the spectral and sensing characteristics of the π-FBG were studied in detail in [11] where for the maximum slow-light sensitivity the optimal grating parameters were obtained. In [12], a review of the current state-of-the-art interrogation principles of πFBG sensors, including a detailed overview of available experimental configurations and their working principles, is presented. Then, resulting advantages and limitations of different architectures are discussed. Aside from the enhanced sensitivity, slow light observed in cavities formed by phase shifted FBGs could help in performance optimization of distributed feedback lasers [13].

The ability to control the propagation group velocity of light will have an impact on the electric field intensity in the material (optical fiber sensor or waveguide). This in turn will change the energy density. This can help to manage linear/nonlinear effects during light propagation. Optical fiber interferometers such as the Mach–Zehnder interferometer (MZI) present another group of devices possibly benefiting from enhancements of the sensitivity and spectral resolution thanks to an increase in the “slow light” group index. Densely integrated photonic circuits such as photonic crystal waveguides (PCWs) can offer advantages at room temperature. However, their near band edge area of operation and susceptibility environment interference present a challenge [14]. Another slow (and fast) light generation method involves interactions between light field and acoustic phonons in a media lattice by utilizing coherent control of acoustic phonons in silica fiber [15].

However, as of today, large-scale silicon photonic integrated circuits incorporating a variety of active and passive functionalities still face considerable challenges. Well-engineered slow light waveguides can provide a viable solution for compact, low-power modulators via enhanced light-matter interactions that can shrink their footprint [16].

A strongly apodized 6.5 mm long FBG written with a femtosecond laser into Er-doped fiber was used to obtain a group velocity of 22 km/s (280 ns group delay) [17]. Good repeatability and flexibility of different phase-shifted FBG inscription techniques have been developed and improved over the past years. The so-called phase mask method, a repeated exposure, or numerous post processing techniques can be also utilized for the formation of a phase-shifting region inside of the grating. An experimentally demonstrated 19.5 ns group delay was observed in a 2 cm long FBG with an index modulation of 1.69 × 10^−3^ in the SMF-28 optical fiber, highlighting the benefits of accurate apodization [18,19,20].

In this paper we report on the theoretical and experimental investigation of a π-phase-shifted FBG (π-PSFBG) by measuring the group delay induced by slow light. Considering fabrication limitations, the design of physical and spectral characteristics is described and modeled. The theory of phase-shifted gratings is utilized for the group delay, transmission, and reflection spectra characterization via simulations in the MATLAB^®^ environment. The simulation results are then compared with experimental data. We have shown that the π-PSFBG operating in its transmission mode produce a pair of delayed (delayed one relative to other) optical pulses. Their relative delay can be controlled by the grating design and by wavelength filtering at the grating input. If the filter is tunable, one can have an integrated tunable delay line for applications in RF photonics, ultra-fast signal processing, and optical communications, where tunable high precision delay lines are highly desirable [21].

## 2. Materials and Methods

The π-PSFBG has been modeled using an algorithm written and implemented for MATLAB programming platform.

**Figure 1:**
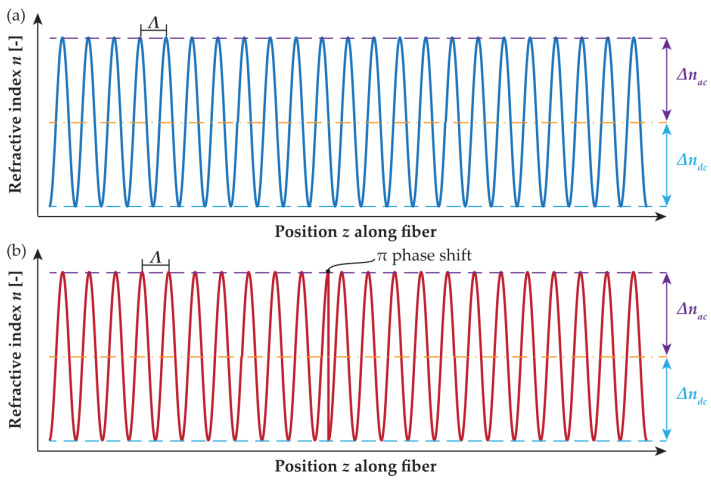
(**a**) Refractive index modulation in a uniform FBG and (**b**) refractive index modulation with inserted π phase shift [22].

To describe the behavior of fiber gratings, we used known and established approach by applying a set of coupled mode equations governing the coupling between the forward and backward propagating modes. As an analytical solution exists only for a lossless uniform case, a piece-wise approach that divides the grating into several subsections is required. We will consider a π-PSFBG to be an optical device realized as a short section of a fiber with the periodic modulation of the fiber core refractive index (depicted in Figure 1) having spatial periods of a sub-micron scale. This condition enables the coupling between forward and backward propagating modes [22].

Calculation of interaction between propagating modes has been represented by a 2 × 2 transfer matrix *T_i_*:

(1) $$\left(\begin{array}{c} F_{i}\\ B_{i} \end{array}\right)=T_{i}\left(\begin{array}{c} F_{i-1}\\ B_{i-1} \end{array}\right)=\left(\begin{array}{cc} T_{11} & T_{12}\\ T_{21} & T_{22} \end{array}\right)\left(\begin{array}{c} F_{i-1}\\ B_{i-1} \end{array}\right),$$

where *F* is forward (incident) and *B* is backward (reflected) mode. Elements of *T_i_* are defined as:

(2) $$T_{11}=\cosh\left(\gamma_{B}\Delta z\right)-i\frac{\sigma }{\gamma_{B}}\sinh\left(\gamma_{B}\Delta z\right),$$

(3) $$T_{12}=-i\frac{\kappa }{\gamma_{B}}\sinh(\gamma_{B}\Delta z),$$

(4) $$T_{21}=T_{12}^{*},$$

(5) $$T_{22}=T_{11}^{*}.$$

where *σ* is the general “dc” self-coupling coefficient, *κ* is the “ac” coupling coefficient, and for each sub-gratings of the length ∆*z γ_B_* = (κ^2^ − σ^2^)^1/2^ if *κ*^2^ > *σ*^2^ or *γ_B_* = *i*(*σ*^2^ − *κ*^2^)^1/2^ if *κ*^2^ < *σ*^2^, respectively. Symbol * denotes complex conjugate.

Coupled-mode equations calculated by using the numerical approach applied to a selected fiber region with relevant boundary conditions were obtained for non-uniform gratings. Here we considered a grating model having a length of *L*, with a forward traveling wave passing through the grating structure, and no backward moving wave for *z* ≥ *L*, such that *F*(*L*) = 1 and *B*(*L*) = 0.

The whole grating was divided along its length *L* into *N* segments represented by uniform FBG sub-gratings of the length *L* ≫ Λ. For each segment, the 2 × 2 transfer matrix coupling the forward and backward electric fields at its output and input at a specific wavelength were calculated. The FBG is a multiple-wave interferometer of the length *L* that introduces a dispersion in the reflected and transmitted signals.

The phase shift matrix *F_ps_* is given as:

(6) $$F_{ps}=\left(\begin{array}{cc} e^{-i\frac{\phi }{2}} & 0\\ 0 & e^{i\frac{\phi }{2}} \end{array}\right),$$

where *ϕ* is the shift in the phase of the grating. The phase shift matrix is introduced as the total response of the whole structure and calculates the relationship between output and input fields in the grating. The total response is calculated as:

(7) $$\left(\begin{array}{c} F_{L}\\ B_{L} \end{array}\right)=T_{N}T_{N-1}\cdots F_{ps}\cdots T_{2}T_{1}\left(\begin{array}{c} F_{0}\\ B_{0} \end{array}\right),$$

where *F*_0_ and *B*_0_ are the input forward and backward amplitudes, and *F_L_* and *B_L_* are the output field amplitudes, respectively [23].

The group delay *τ_g_* is the derivative of *θ_ρ_* with respect to the angular frequency ω:

(8) $$\tau_{g}=\frac{d\theta_{p}}{d\omega }=\frac{d}{d\omega }\arg\left(r\right)=\frac{-\lambda^{2}}{2\pi c}\frac{d\theta_{p}}{d\lambda }=\frac{n_{g}L}{c},$$

where *θ_ρ_* is the phase of the field, *r* is reflection*, n_g_* is the group index, and *c* is the speed of light in the free space.

In many applications, the usable bandwidth of optical fiber is limited due to the temporal dispersion of the optical pulse that leads to an inter symbol interference and subsequently to information loss. For long-distance applications, the use of single mode fibers with only one supported propagating mode is therefore preferable. Still, the group velocity dispersion needs to be considered even in single mode fibers [24]. The group velocity can be defined by using the group index *n_g_* as follows:

(9) $$v_{g}=\frac{c}{n_{g}},$$

and the signal wavelength related to dispersion *D*:

(10) $$D=\frac{-2\pi c}{\lambda^{2}}\beta_{2},$$

where *D* is a dispersion coefficient and *β*_2_ is a group velocity dispersion coefficient responsible for the pulse broadening defined as:

(11) $$\beta_{2}=\frac{d^{2}\beta }{d\omega^{2}}.$$

In the grating design process, the following parameters were used: *n_eff_* = 1.46; Φ = π; Δ*n* = 5 × 10^−4^; and Λ = 0.53185.

The following optical definitions need to be introduced to properly evaluate the slow light performance. The group velocity is the first-order differential of the dispersion curve. The group index is characterized as the slow-down factor, describing the decrease in the group velocity compared to its value in vacuum. Respecting the foreseen practical applications of slow light, it is desirable to hold a constant value of the group index across a wide bandwidth, and a value of the group velocity dispersion GVD (second-order dispersion) that is as small as possible.

To evaluate the slow-light generation, several optical several optical parameters need to be considered. To achieve ultra-slow light, the slope of the dispersion needs to be close to zero. Considering the practical applications of slow light, the group velocity *v_g_ = dω/dk* should be kept constant across the wide bandwidth range, and the group velocity dispersion GVD = *d*^2^*ω/dk*^2^ should remain as small as possible. When the goal is a “strong” slow light effect, the group index *n_g_* should be high. Unfortunately, any rise in *n_g_* is accompanied by an undesirable narrower operational bandwidth. Therefore, to evaluate the slow light efficiency of the given structure, a normalized delay–bandwidth product (NDBP = *n_g_*(Δ*ω*/*ω*_0_)) as the compromise between *n_g_* and the bandwidth is introduced [25].

## 3. Results

### 3.1. First π-PSFBG Design

The slow light resonance located in the phase shifting area of the fiber Bragg grating is related to a large group delay, and together with the high transmissivity they both belong to the two the most important features when designing a π-PSFBG based sensor or a time delay element. However, a number of factors is involved in achieving desired properties and they require careful evaluation of the different parameters that impact the final structure design.

To properly model the desired phase shift and to study the formed cavity, first, we have focused on a length parameter while keeping the other parameters fixed. A favorable increase in the calculated the group delay was a result of the accurate design of multiple complementary features. A half spatial period-wide discontinuity inserted when inscribing the grating should translate into a π phase shift. This was numerically modeled and confirmed. By numerically examining the effect of the length of the grating on the amount of group delay, we observed that the phase-shift decrease in the wavelength span was accompanied by higher values in the reflectivity and group delay (when compared to a basic π-PSFBG design, a design with a single-phase shift placed in the middle of the uniform FBG). The observed changes are important for evaluation of the grating real-world inscription method accuracy.

For the experimental evaluation of the group delay generated by the π-PSFBG, we designed a grating that was manufactured by FiSens GmbH (Braunschweig, Germany) to operate in its reflection mode. For the grating’s phase shifting area, we predicted a group delay value of 357 ps and a sufficient spectral width of an area “generating” slow light.

In our design, we assumed a relatively high value (357 ps) of time delay to be generated in a 5 mm long grating with a refractive index modulation equal to 5 × 10^−4^. The phase shift equal to one π was placed in the center of the structure with a given spatial period and the designed central wavelength of 1553 nm. The structure was written into a standard Corning^®^ SMF-28 telecommunications fiber without the use of methods to increase photosensitivity using a point-to-point method by a femtosecond laser with no further details provided by the manufacturer.

**Figure 2:**
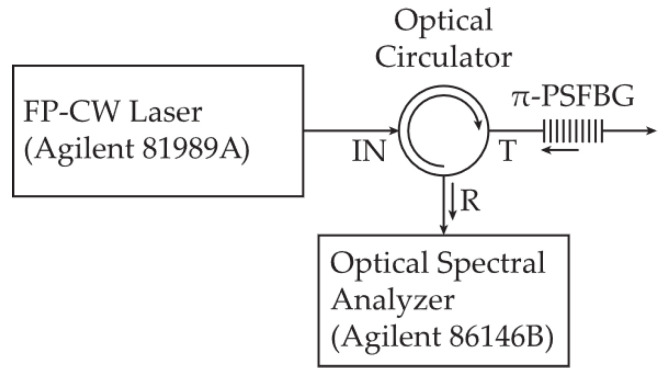
Measurement setup for investigation of the first π-PSFBG that was designed to operate in its reflection mode.

The investigation of the manufactured π-PSFBG that was designed to work in its reflection mode was conducted at the University of Strathclyde using the setup shown in Figure 2. A wavelength tunable narrow-linewidth (100 kHz) Fabry–Pérot (FP) laser (Agilent 81989A with a built-in wavemeter for accuracy of ±20 pm) was used at the input (IN) of an optical circulator (OC).

**Figure 3:**
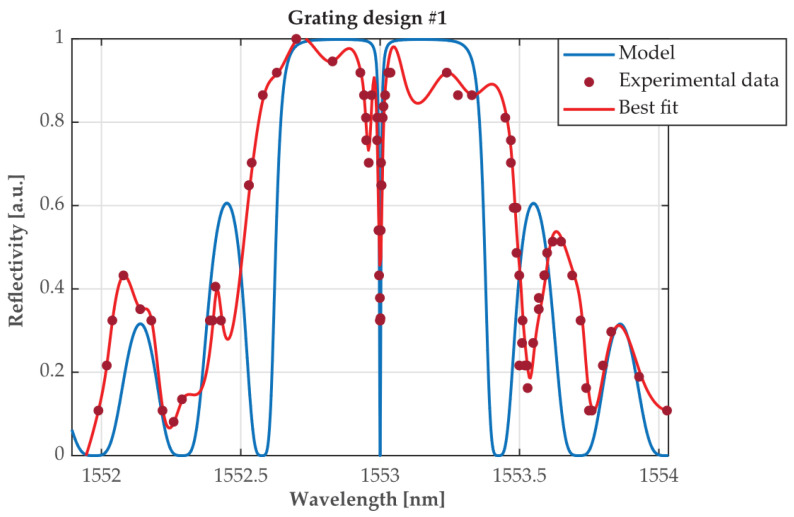
The modeled reflectivity spectra of a first π-PSFBG design (blue line) compared to experimentally obtained values (red dots) and their best fit (red line), respectively.

The output-T of the OC was connected to the π-PSFBG input. The signal reflected back by the π-PSFBG, after transversing the OC, was read via its port-R observed on the optical spectrum analyzer, Agilent 86146B, with a max resolution of 60 pm. No averaging or filtering was used. The results of the investigation are shown in Figure 3, where the modeled reflectivity spectra of the π-PSFBG are shown as a blue line and the experimentally obtained values are shows as red dots. One can observe some discrepancies in the interval further away from *λ_R_* = 1553 nm. Since the spec. resolution of the OSA is 60 pm and the Fabri-Pérot tunable cw laser has a wavelength tuning accuracy of ±20 pm, the observed discrepancies could be attributed to grating’s imperfections resulting from the manufacturing processes rather than to the measurements accuracy.

The observed spectra “indentation” seen on experimental data, when compared to modeling results, could also be attributed to fabrication impairments. On the other hand, there is a very-good agreement between the values of the measured and modeled coefficient of reflection surrounding the central wavelength (*λ_R_* = 1553 nm). Measurements of the grating’s spectral characteristic show that the resulting “central wavelength reflection passband is spectrally too narrow” (less than 10 pm which is well below the resolution limit of 60 pm of the used OSA (Agilent 86146B). We found that not enough “measurable” optical power was reflected at the π-PSFBG output for conducting successful measurements for determining the group delay introduced by the π-PSFBG at *λ_R_*. The grating’s spectral width was designed to be less than 10 pm because this value was expected as necessary to generate the modeled group delay of 357 ps.

### 3.2. Second π-PSFBG Design

Based on the experience, a second grating was designed with modified length and index of modulation. These modifications were in general terms consulted with the manufacturer in order to better understand the challenges of the fabrication process.

The main challenges in the π-PSFBG fabrication process were the grating’s relatively small dimensions and the accuracy of placing the π-phase shift in the FBG center. When writing the selected spatial period directly related to the central wavelength, i.e., the practical implementation of the phase shift, the first-half period was skipped and second only was implemented. The main challenge was to secure the position of the displacement in the center of the grating. At the same time, the design process had to carefully consider the trade-off between the achievable and measurable time delays generated by the fabricated grating because this is directly influenced by achievable production parameters, such as the grating length and refractive index modulation depth.

The manufacturing error, which is the consequence of a writing process of the grating to the fiber, was reassessed when designing this second grating. This was performed based on both the experience with the first design and the obtained experimental results.

The modified approach relied on the simulation of a chirped grating with the chirp vector having a pseudo-random characteristic. We used this approach to simulate manufacturing errors; however, instead of the required chirp function, we generated the vector of pseudo-random values to disturb a spatial period across the grating. We used a built-in MATLAB function to generate random scalar values drawn from the uniform distribution in the interval (0,1). Allowing for varied possibilities of adapting values, in other words, allowing for the possibility of simulating an imperfect inscription process, this method helped with selecting the final reflectivity and group delay spectra. However, the main challenge of this approach was to simulate any dependence on a single specific external influence, such as temperature fluctuations, mechanical vibrations, an uneven displacement of the fiber in the femtosecond laser writing method, and manufacturing imperfections of a used fiber (micro bends, fiber symmetry disturbances, or material defects). Alternatively, by using a simplified model, all these different influences can be superimposed and viewed as a “resulting random phenomenon” characterized by values of the used vector.

Once this design was completed and the π-PSFBG was fabricated, our preliminary simulation of the group delay showed that its value was approximately 20 ps. The results also indicated that this grating is expected to have a wider slow light spectral passband when compared to the first grating design and that enough optical power should reach the grating’s output at the transmission port T.

**Figure 4:**
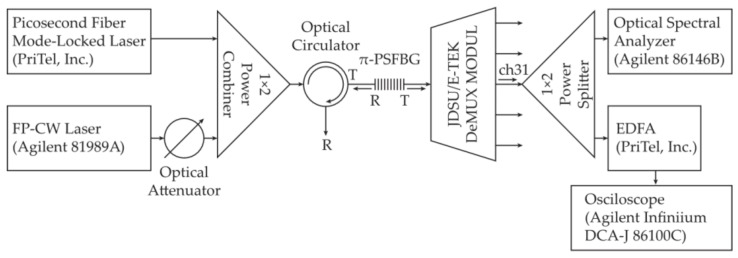
Experimental setup of a phase-shifted π-PSFBG. To filter out the unwanted signal at the grating output, a ch31 filter (on a 100 GHz ITU grid) of a JDSU/E-TEK DeMUX module was inserted.

In our experimental investigation, we opted for the experimental setup depicted in Figure 4. At the input, we used a sech^2^ optical laser pulse (2 ps FWHM and 1.4 nm spectral width) generated by a tunable erbium-doped fiber actively mode-locked laser (PriTel, Inc., Naperville, IL, USA). The laser output was spectrally tuned to overlap with the designed central wavelength transmission region of the investigated π-PSFBG. The laser pulse, after passing the π-PSFBG, is delayed due to its interaction with the grating. The largest time delay is for the designed grating central wavelength *λ_cd_* = 1552.42 nm.

To enable measurements of the generated delay at the transmission output of the π-PSFBG, a channel ch31 of a 100 GHz JDSU/E-TEK WDM DeMUX with a central wavelength of 1552.52 nm was placed at the π-PSFBG output (see Figure 4). The channel ch31 was selected because its passband width is narrow enough (0.8 nm) to filter out unwanted frequencies at the output but at the same time also allows for a passage of a portion of the laser pulse adjacent to the central wavelength *λ_cd_*. (This will serve as a “reference” to determine the relative delay Δ*τ* the grating introduces between the exiting pulse at the designed central wavelength λ*_cd_* and at a selected adjacent wavelength *λ*.)

The optical signal exiting the π-PSFBG was then 50:50 split by a 1 × 2 optical power splitter (see Figure 4). One end was connected to an optical spectrum analyzer and the second end, via an erbium doped fiber amplifier, to a digitizing oscilloscope (Agilent Infiniium DCA-J 86100C equipped with an optical sampling head having ~10 ps resolution).

**Figure 5:**
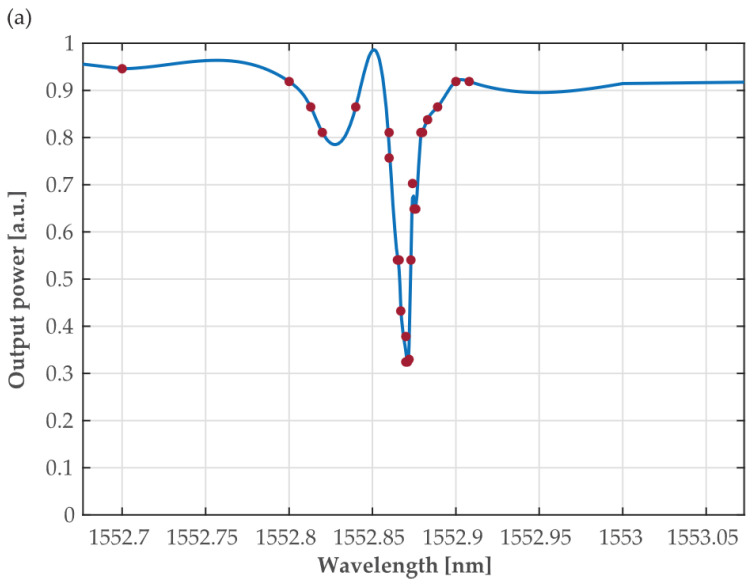

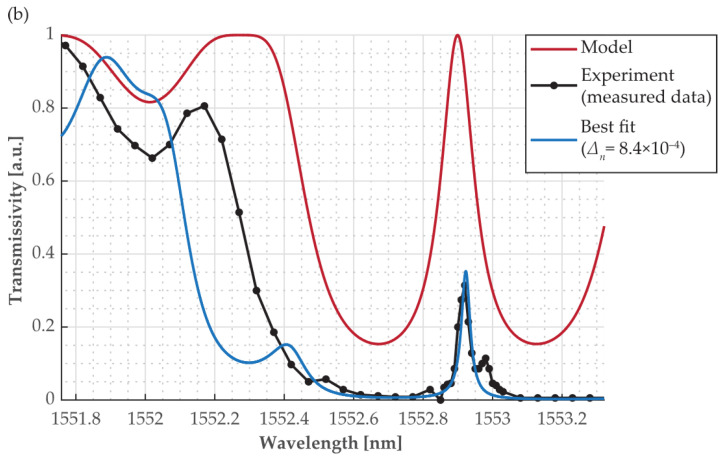
(**a**) Measured spectral reflectivity of the 2.8 mm π-PSFBG. The red dots represent experimental data, and the blue line is the best fit; (**b**) spectral characteristics of the 2.8 mm π-PSFBG. Modeled (red line), measured data (black), and the blue line is data best fit, respectively. Notice a small difference between the value of the designed central wavelength *λ_cd_* = 1552.9 nm and its measured value *λ_cm_* = 1552.92 nm. The measured lower transmissivity is a result of the omission of a loss coefficient in the model.

At first, the measurements were taken without the presence of the channel ch31 filter, and we used a wavelength tunable cw narrow-linewidth (100 kHz) Fabry–Pérot laser (FP) at the π-PSFBG input. The results are shown in Figure 5.

The π-PSFBG spectral reflectivity measured at the OC port-R is shown in Figure 5a, where red dots are experimental data and the blue line is their best fit.

The π-PSFBG spectral transmissivity measured at the OC port-T is shown on Figure 5b where black dots are experimental data and the best fit is the blue line. For comparison, the red line shows modeled spectral transmission of the π-PSFBG. Notably, there is a good agreement between the experimentally measured wavelength value of the transmission and the reflection pass-band peak, respectively (see Figure 5a,b); both values are ~1552.92 nm). A small difference can be seen between the value of the designed transmission central wavelength *λ_cd_* = 1552.9 nm and its measured value *λ_cm_* = 1552.92 nm. This difference between the theoretical prediction and the experimentally measured value is influenced by two factors. One, our model does not account for a loss parameter and the manufactured grating is also affected by various imperfections from the manufacturing processes. Two, the measurements are influenced by losses in the devices used in the experimental setup for the π-PSFBG performance investigation.

**Figure 6:**
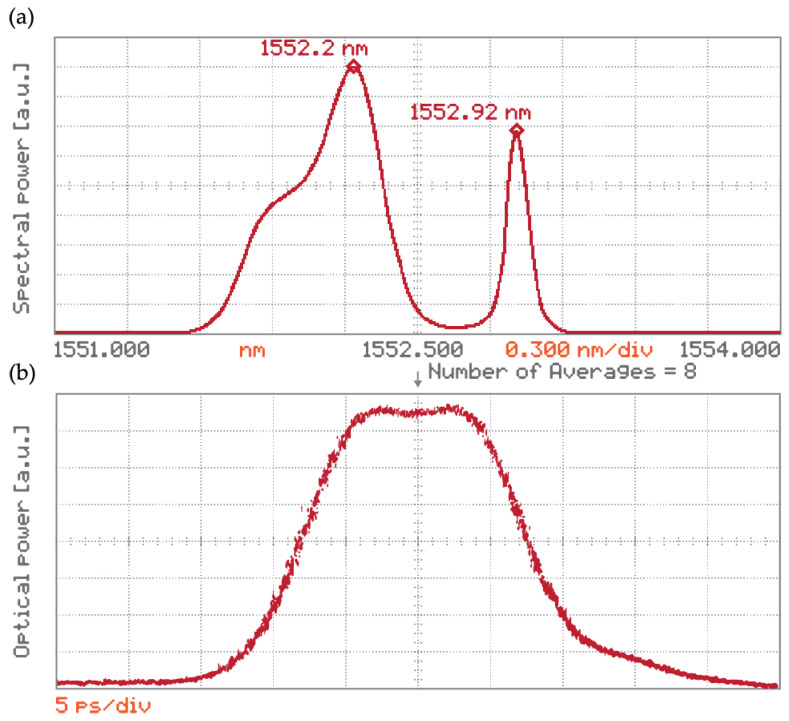
(**a**) π-PSFBG output in the spectral domain observed on the optical spectrum analyzer (note two wavelength peaks at *λ* = 1552.2 nm and *λ_cm_* = 1552.92 nm); (**b**) grating output in the time domain as seen on a digitizing oscilloscope with an ultra-fast optical sampling head.

Next, the FP cw laser was replaced by the PriTel laser described earlier and spectrally tuned to overlap with the measured central wavelength *λ_cm_* = 1552.92 nm found in the previous experiment. The DSU/E-TEK WDM DeMUX ch31 was inserted back into the setup (Figure 4) The impact of the ch31 filtering on the π-PSFBG transmission output is shown in Figure 6a. To experimentally find the value of the relative delay Δ*τ* the π-PSFBG introduces between pulses, say at *λ* = 1552.2 nm and *λ_cm_* = 1552.92 nm (see Figure 6a), the π-PSFBG output was fed into a digitizing oscilloscope with an ultrafast optical sampling head via an erbium doped fiber amplifier (EDFA), see Figure 4. The result is shown in Figure 6b.

**Figure 7:**
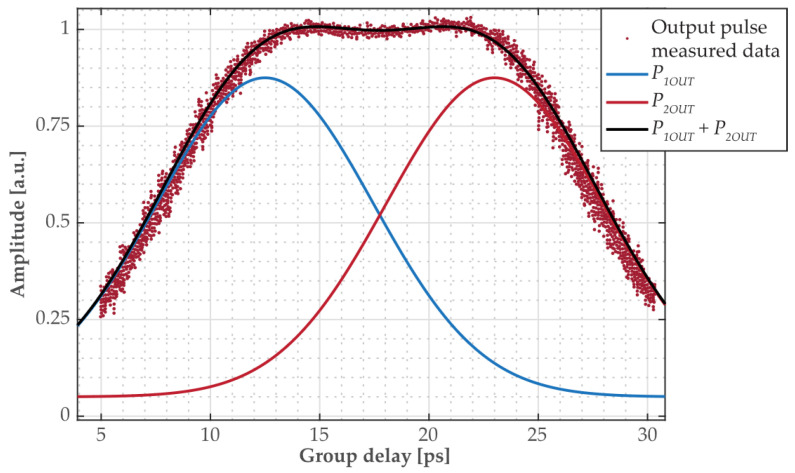
Time domain characteristics of output impulse (red data points). Apart from the widening caused by dispersion, the output impulse is composed of two impulses experiencing different amounts of the group delay.

To better determine the relative time delay seen on the oscilloscope, we assumed the curve in Figure 6b to be the result of the superposition of two partially overlapping sech^2^ pulses, of which spectra are shown in Figure 6a and have wavelengths of *λ* = 1552.2 nm and *λ_cm_ =* 1552.92 nm, respectively. Such a scenario was simulated, and the results are shown in Figure 7. From here, the relative delay Δ*τ* was estimated as Δ*τ* = 10.5 ps.

**Figure 8:**
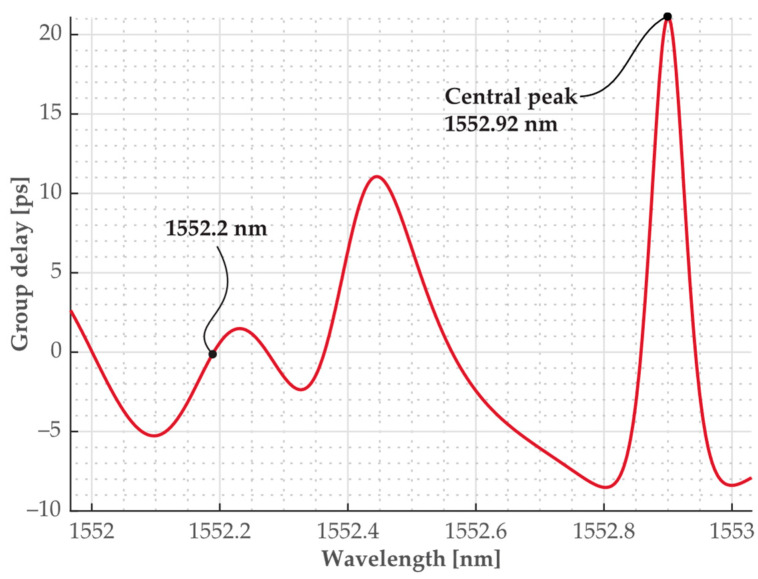
Calculated/modeled relative group delay as a function of wavelength generated by the grating at its transmission output. (For *λ* = 1552.2 nm and *λ_cm_* = 1552.92 nm see Figure 6a).

Next, we compared the experimentally obtained value of Δ*τ* with the value Δ*τ_d_* predicted by the π-PSFBG grating design. The group delay Δ*τ_d_* as a function of a wavelength *λ* is plotted in Figure 8. The relative delay for the wavelengths *λ* = 1552.2 nm and *λ_cm_* = 1552.92 nm found from Figure 8 is Δ*τ_d_* = 19 ps and is in a reasonable agreement with the experimentally measured value Δ*τ* = 10.5 ps. The difference between the theoretical prediction of the group delay Δ*τ_d_* based on the simulation results in Figure 8 and the value of Δ*τ* determined from measurements from Figure 6 and Figure 7 is caused by a number of factors. One, the developed model does not account for the loss parameter. Two, the manufactured grating is affected by imperfections during the fabrication. Three, the conducted measurements are affected by a bandwidth limited oscilloscope (Agilent Infiniium DCA-J 86100C) equipped with an optical sampling head of ~10 ps resolution. Four, an accurate determination of wavelengths by the OSA was also a contributing factor.

## 4. Discussion and Conclusions

We have designed, numerically simulated, and experimentally investigated the spectral characteristics of two π-phase-shifted fiber Bragg gratings (π-PSFBGs) inscribed in the standard telecom fiber for slow light generation. The first grating was designed to operate in the reflection mode with a central wavelength of *λ_R_* = 1553 nm and a pass band width of less than 10 pm. Such a narrow spectral region provided only a limited optical-output power at the grating-reflection output for use in measurements. The impact of fabrication imperfections was also analyzed and taken into account for optimization of the grating second design. The goal was to limit fabrication errors to improve the grating’s spectral behavior, its temporal performance, and also widen the spectral interval for slow light generation by the π-PSFBG operating in its transmission mode at a central wavelength of *λ_cd_* = 1552.9 nm. This π-PSFBG was then fabricated, and its parameters were experimentally investigated. The measured central wavelength value was found to be *λ_cm_* = 1552.92 nm, which is in a good agreement with the model prediction. The measured group delay was found to be Δ*τ* = 10.5 ps compared to Δ*τ_d_* = 19 ps as predicted by the design model.

The grating’s spectral reflectivity was also measured. We noted a very good agreement between the experimentally measured wavelength values of the transmission and reflection pass-band peaks, respectively.

In conclusion, this type of the π-PSFBG can be designed and used to generate group delays from a few to hundreds of ps, depending on application needs. We have shown that a pair of delayed optical pulses can be produced by the π-PSFBG operating in transmission mode. Their relative delay can by controlled by the grating design and further fine-tuned by a wavelength filtering at the grating input. If this filtering is a tunable wavelength filter, an integrated tunable delay line can be achieved. Also, as shown elsewhere, FBG sensors based on slow light offer a higher sensitivity, wider dynamic range, and better resolution in comparison to conventional FBG sensors.

## Data Availability

Data are contained within the article.
